# Naltrexone for Acute Management of Compulsive Sexual and Obsessive Symptoms in a Patient With a Neurodevelopmental Disorder

**DOI:** 10.1155/crps/9442957

**Published:** 2026-06-09

**Authors:** Eric Constantin, Moonpreet S. Deol, Jeffrey Yaun, Hardeep Singh, Oliver M. Glass

**Affiliations:** ^1^ Graduate Medical Education, Northeast Georgia Medical Center, Gainesville, Georgia, USA, nghs.com; ^2^ Psychiatry, Northeast Georgia Medical Center, Gainesville, Georgia, USA, nghs.com; ^3^ Medical College of Georgia, Augusta University and University of Georgia Medical Partnership Campus, Athens, Georgia, USA, gru.edu

**Keywords:** attention-deficit/hyperactivity disorder, bipolar disorder, compulsive sexual symptoms, intellectual developmental disorder, naltrexone, obsessive–compulsive disorder, sexual obsessions

## Abstract

Compulsive sexual behavior disorder (CSBD) is characterized by persistent difficulty controlling sexual urges and behaviors associated with marked distress or impairment. Population‐based data indicate that ~8.6% of US adults experience distress related to difficulty controlling sexual urges, feelings, and behaviors, while large international screening studies suggest that ~5% of individuals may be at high risk for CSBD, yet only a minority seek treatment. Diagnostic classification remains challenging in early or evolving presentations and in patients with complex psychiatric and neurodevelopmental comorbidities. The International Classification of Diseases, 11^th^ Revision (ICD‐11) requires a 6‐month symptom duration for formal diagnosis and specifies exclusion when symptoms are better explained by another mental disorder, substance use, or medication effects. Here we describe a man in his 20 s with bipolar I disorder, attention‐deficit/hyperactivity disorder, anxiety, and intellectual developmental disorder (IDD), along with medical comorbidities, who was hospitalized for severe intrusive sexual thoughts associated with distress and safety concerns. Phenomenology suggested overlap between obsessive–compulsive disorder (OCD) with sexual obsessions and an emerging CSBD‐like syndrome, with ego‐dystonic intrusive cognitions alongside strong gratification‐driven urges and restraint primarily driven by anticipated negative consequences. Given symptom severity and imminent risk of behavioral enactment, the presentation was conceptualized as an early CSBD‐like syndrome despite not meeting the ICD‐11 duration criterion. Symptoms remained acutely refractory despite multiple psychotropic optimizations; the most temporally associated improvement occurred after initiation of naltrexone, with marked improvement within 5 days and resolution of intrusive sexual thoughts by hospital day 16. Although concurrent medication changes preclude attribution to naltrexone monotherapy, the rapid response supports further investigation of reward‐ and impulse‐targeted interventions for severe subthreshold presentations and highlights the need for clearer diagnostic frameworks and evidence‐based guidance for acute management of diagnostically ambiguous compulsive sexual and sexual obsessive syndromes.

## 1. Introduction

Population‐based data indicate that ~8.6% of US adults experience distress related to difficulty controlling sexual urges, feelings, and behaviors [1, 2]. Large international screening studies further suggest that ~5% of individuals may be at high risk for compulsive sexual behavior (CSBD); however, only a minority of affected individuals seek treatment [3]. CSBD is defined as a persistent pattern of failure to control intense, repetitive sexual impulses or urges resulting in repetitive sexual behavior and is most commonly reported in males [4, 5]. According to the International Classification of Diseases, 11^th^ Revision (ICD‐11) diagnostic guidelines, these symptoms must persist for at least 6 months for a formal diagnosis to be established [5, 6]. Importantly, ICD‐11 specifies that CSBD should not be diagnosed when the sexual symptoms are better explained by another mental disorder (e.g., a manic episode), substance use, or medication effects [4, 5]. At present, no diagnostic category exists for individuals with clinically significant symptoms that fall below this duration [5–7]. Such presentations may therefore be conceptualized as subthreshold or prodromal CSBD, particularly when symptoms are severe, progressive, or acutely refractory to early intervention [7].

This condition often leads to profound implications in patients’ social and professional lives [8]. For instance, marriages or partnerships can deteriorate because of the covert or excessive aspects of sexual behaviors. People with CSBD might face a heightened risk of acquiring a sexually transmitted infection due to unsafe or frequent sexual activities. These compulsive sexual urges can also disrupt their professional lives, potentially causing job loss due to their fixation on sexual activities or resulting in legal issues from inappropriate actions like public indecency or engaging in illegal services [5]. In a study looking at 36 individuals with CSBD, 42% reported that repetitive sexual behaviors had affected their relationships or marriages, 25% reported that their sexual behaviors were affecting their work, and 47% felt that the repeated sexual urges adversely affected their social lives [9].

Several conditions are often seen alongside CSBD as comorbidities. For example, individuals with CSBD show a higher prevalence of clinical conditions that involve significant emotion regulation challenges [10–12]. CSBD is strongly associated with attention‐deficit/hyperactivity disorder (ADHD), with a reported prevalence of ~17% [10]. In comparison, the prevalence of ADHD in US adults is 4.4%, showing a significant difference in the CSBD population versus non‐CSBD [13]. ~40% of those with CSBD have been diagnosed with major depressive disorder at some point in their lives. Additionally, 20% experience moderate to severe depression, 21% have had an adjustment disorder at some time, and 6% have been diagnosed with bulimia nervosa (as compared to only 0.3% prevalence in the total US population) [11, 14]. Hypersexuality is also very common in patients with bipolar disorder, as it occurs in about 25% to 80% of bipolar patients [15, 16]. Hypersexuality is one of the most common symptoms during manic and hypomanic episodes in bipolar patients. These patients with CSBD are also at a higher risk of comorbid substance use disorders [17, 18].

A critical diagnostic consideration in the differential evaluation of compulsive sexual behavior is obsessive–compulsive disorder (OCD) with sexual obsessions [19]. In OCD, intrusive sexual thoughts or images are experienced as unwanted, distressing, and inconsistent with the individual’s values and are typically accompanied by efforts to suppress or neutralize these thoughts through mental or behavioral compulsions [19–21]. Co‐occurrence of OCD and CSBD is not uncommon, with ~3.8–5.6% of individuals with OCD also meeting criteria for CSBD [19]. However, robust epidemiologic data describing the prevalence of OCD among individuals with CSBD are currently lacking, and no large population‐based studies have definitively established this relationship. When both disorders are present, clinical presentations are often more complex and associated with greater psychiatric comorbidity. Careful phenomenological assessment is therefore essential in distinguishing between these entities and guiding appropriate clinical management.

At present, no pharmacologic treatments for CSBD have received United States Food and Drug Administration approval [22]. However, the World Federation of Societies of Biological Psychiatry (WFSBP) guidelines identify selective serotonin reuptake inhibitors and naltrexone as pharmacologic options, with selection guided by symptom profile and clinical context [23]. The overall concrete evidence base remains limited and conflicting [24], particularly for acute and subthreshold presentations, which is especially consequential in cases requiring rapid stabilization to preserve safety and care in the least restrictive environment.

These diagnostic and therapeutic challenges underscore the clinical importance of recognizing subthreshold, prodromal, and acutely refractory presentations of CSBD. The following case illustrates the complexity of diagnostic formulation and treatment planning in a patient with severe, functionally impairing compulsive sexual symptoms occurring in the context of multiple psychiatric comorbidities and significant safety concerns.

## 2. Case Presentation

A male patient in his 20s presented to the emergency department with a past medical history of bipolar I disorder, ADHD, anxiety, intellectual developmental disorder (IDD), type 2 diabetes mellitus, gastroesophageal reflux disease, and hypertension. He reported a 2–3‐month history of progressively worsening intrusive sexual thoughts primarily centered on his mother, accompanied by intense urges to engage in sexual behaviors. He expressed significant distress and fear that he might act on these urges and admitted himself voluntarily for inpatient psychiatric hospitalization.

Beyond the intrusive thoughts, the patient described recurrent sexual fantasies and strong urges toward masturbation, often involving imagined sexual scenarios, which he found difficult to resist. He reported spending substantial time preoccupied with these urges and described mental rehearsal, planning, and internal conflict surrounding potential behavioral enactment. Although he had not acted on these impulses, he acknowledged a drive toward gratification, with restraint primarily related to anticipated negative consequences and moral and social prohibitions. He endorsed significant guilt and shame related to the taboo nature of these thoughts and urges. Symptom intensity increased in closer proximity to his mother. While the intrusive cognitions were experienced as distressing and unwanted, consistent with obsessive phenomena, the accompanying urges and pursuit of gratification demonstrated features more consistent with compulsive sexual behavior. He denied any prior sexual experiences or romantic relationships.

Importantly, the patient did not display or report cardinal features of a manic episode. Aside from increased hypersexuality and mild sleep disturbance, which were clinically attributed to situational distress, there were no symptoms suggestive of mania or hypomania. In contrast, he exhibited notable depressive symptoms, which were related either to the psychological distress of the presentation or to his underlying bipolar I disorder.

At the time of evaluation, the patient resided with his adoptive parents, who were also his legal guardians, and worked alongside his father. He reported limited social engagement and was financially dependent on his family. Given his limited social connectedness, absence of intimate relationships but noted desire for them, and significant psychiatric comorbidity, concerns were raised regarding psychosocial and developmental contributors to his presentation.

During hospitalization, the patient completed the Sexual Compulsivity Scale [25, 26] (Table 1), endorsing the highest level of severity across all items (40/40). Such an extreme score should be interpreted with caution and may reflect the severity of underlying psychiatric traits rather than serving as a definitive diagnostic marker. The validity of this result is further complicated by potential confounding from comorbid neurodevelopmental factors, obsessive–compulsive features, limited insight, and concrete response styles, all of which may influence self‐report measures. Because questionnaire data alone is insufficient to establish a diagnosis of CSBD, the scale findings were considered supportive but not determinative within the overall clinical formulation.

**Table 1:** Sexual compulsivity scale as responded to by patient.

Question	Score (1 = not at all like me; 2 = slightly like me; 3 = mainly like me; and 4 = very much like me)
My sexual appetite has gotten in the way of my relationship.	4
My sexual thoughts and behaviors are causing problems in my life.	4
My desires to have sex have disrupted my daily life.	4
Sometimes I fail to meet my commitments and responsibilities because of my sexual behaviors.	4
I sometimes get so horny I could lose control.	4
I find myself thinking about sex while at work.	4
I feel that sexual thoughts and feelings are stronger than I am.	4
I have to struggle to control my sexual thoughts and behavior.	4
I think about sex more than I would like to.	4
It has been difficult for me to find sex partners who desire having sex as much as I want to.	4
	Total: 40/40

At home, the patient was maintained on fluoxetine, clonidine, guanfacine, trazodone, and olanzapine. Prior to admission to the inpatient psychiatric unit, olanzapine was held due to concerns regarding limited clinical benefit. Given the patient’s underlying diagnosis of bipolar I disorder, ongoing treatment with psychotropic medication was indicated to reduce the risk of relapse. At the time of presentation, there was additional concern that the patient may have been entering a depressive phase, which was contributing to the severity of his symptoms. Accordingly, a decision was made to initiate alternative mood augmentation with risperidone. Risperidone was selected for its relatively favorable metabolic profile and was carefully titrated throughout the hospital stay.

Risperidone dosing was adjusted in response to the patient’s reported depressive symptoms, guilt, remorse, and sleep‐onset difficulties, which were initially conceptualized within the context of bipolar I disorder. As hospitalization progressed, these affective symptoms may have been driven primarily by psychological distress related to the compulsive sexual symptoms. Upon admission, an effort was made to simplify the patient’s alpha‐2 adrenergic agonist regimen by consolidating guanfacine and clonidine to clonidine alone, which was subsequently titrated and optimized during the hospital stay.

Concurrently, carbamazepine was initiated to introduce an alternative mechanism for mood and impulse modulation (Table 2), given concern that affective dysregulation may have been amplifying the patient’s compulsive sexual symptoms. During the hospitalization, the dose of fluoxetine was also titrated to reduce libido, target mood, and address the obsessive component of the presentation, with the understanding that full clinical response might not be observed until after discharge.

**Table 2:** Medications taken before and after symptom improvement.

Medication	Home regimen (mg daily)	Admission regimen (mg daily)	Regimen during improvement (mg daily)	Discharge regimen (mg daily)
Fluoxetine	20	60	60	60
Clonidine	0.1	0.1	0.1	0.2
Trazodone	100	100	100	50
Guanfacine ER	4	—	—	—
Risperidone	—	2	2	5
Olanzapine	10	—	—	—
Carbamazepine	—	200	—	—
Naltrexone	—	—	50	50

By hospital day two, carbamazepine demonstrated no meaningful effect on mood or behavioral impulses, and naltrexone was added to the regimen. Given the persistence of severe urge‐driven symptoms, ongoing safety concerns, and the need for rapid stabilization, naltrexone was added to target compulsive and reward‐driven sexual urges (Table 3). Throughout the hospitalization, medications were systematically optimized to maintain stability and facilitate rapid symptomatic improvement prior to safe discharge, particularly considering ongoing safety concerns, as both the patient and his mother reported not feeling safe with an immediate return home, and alternative discharge arrangements required consideration.

**Table 3:** Summary of pharmacologic studies evaluating Naltrexone and SSRIs in compulsive sexual behavior.

Study	Study type	Results
Rodriguez‐Manzo and Fernandez‐Guasti [27]	Animal experimental study (*n* is not applicable)	Treatment with opioid antagonists (naloxone and naltrexone) in rodent models produced dose‐dependent effects on sexual behavior, including shortened ejaculation latency and increased copulatory activity. Clinical implications for CSBD remain uncertain.
Grant and Kim [28]	Case report (*n* = 1)	A patient with kleptomania and compulsive sexual behavior who did not respond to fluoxetine demonstrated a reduction in stealing and sexual urges following treatment with naltrexone.
Raymond et. al [29]	Case report (*n* = 2)	Two patients with compulsive sexual behavior were treated with SSRIs and naltrexone; SSRI response was variable, while one patient demonstrated improvement with naltrexone.
Ryback [30]	Retrospective case series (*n* = 21)	15 of 21 participants treated with naltrexone demonstrated reduction in hypersexual behaviors, while six showed limited or no response.
Wainberg et al. [31]	Randomized, double‐blind, and placebo‐controlled trial (*n* = 28)	Treatment with citalopram was associated with a reduction in compulsive sexual behaviors compared to placebo. Improvements were partial and varied across outcome measures.
Gola and Potenza [32]	Case series (*n* = 3)	Treatment with paroxetine was associated with reduced pornography use and anxiety; however, the emergence of new dyadic sexual behaviors was reported after 12–14 weeks.
Savard et al. [33]	Feasibility study (*n* = 20)	Participants treated with naltrexone demonstrated decreased Mean Hypersexual Disorder: Current Assessment Scale (HD:CAS) scores (10.9 to 4.95 over 4 weeks); a subset showed minimal or no improvement.
Sultana and Sahib Din [34]	Case report (*n* = 1)	Reduction in compulsive sexual behaviors was reported following initiation of naltrexone in a patient with comorbid alcohol use disorder.
Singh et al. [35]	Case report (*n* = 1)	Improvement in compulsive sexual behavior and comorbid depressive symptoms was described following treatment with fluoxetine.

Although multiple pharmacologic changes were implemented, the most robust and temporally associated clinical improvement occurred following initiation of naltrexone. Over the subsequent 5 days, the patient demonstrated marked improvement in mood with complete resolution of intrusive sexual thoughts. On hospital day seven, carbamazepine was discontinued, and later during his stay trazodone was tapered to reduce sedation burden and allow clearer assessment of naltrexone’s therapeutic effect. The patient continued to report sustained remission of sexual urges and intrusive thoughts on naltrexone in combination with his remaining psychotropic regimen.

By hospital day 16, he was discharged in stable condition, denying ongoing sexual thoughts or urges. His discharge medications included naltrexone, risperidone, fluoxetine, clonidine, and trazodone. Postdischarge follow‐up data were unavailable, as the patient did not continue care within our health system.

## 3. Discussion

Considering the patient’s psychiatric history of bipolar disorder, IDD, anxiety, and ADHD, as well as his psychosocial context characterized by limited social contacts, restricted internet access, and the absence of significant female relationships outside of his mother, the clinical presentation was inherently complex. The presence of IDD further contributed to concrete thinking and limited insight [36], although this was accompanied by a notable degree of openness and transparency, which proved helpful for obtaining an accurate clinical history.

Although the case was approached through the lens of CSBD‐like symptoms, the presentation did not meet ICD‐11 diagnostic criteria, as symptoms had not been present for the required 6‐month duration, and there was no evidence of enacted repetitive sexual behaviors [6]. While the patient expressed sexual desire, he consistently refrained from acting on these urges due to awareness of potential negative consequences. Importantly, the clinical picture also suggested a possible co‐occurrence or alternative diagnosis of OCD with sexual obsessions. This distinction rests on phenomenological features, particularly whether the symptoms are primarily anxiety‐driven versus gratification‐driven, and requires individualized, case‐by‐case assessment. The patient displayed features of both conditions; however, treatment was directed primarily toward sexual impulsivity to prevent progression to behavioral enactment. No clear precipitating sexual trauma or external trigger was identified. Notably, had symptom severity and safety concerns been lower, management would likely have favored close outpatient monitoring to observe symptom progression, as such symptom clusters can be transient [1, 19, 37]. However, there remains a significant lack of clinical guidelines addressing acute management of subthreshold CSBD and acute OCD with sexual obsessions.

In accordance with ICD‐11 diagnostic criteria, CSBD should not be diagnosed when sexual symptoms are better explained by another mental disorder (e.g., manic episode), substance use, or medication effects [6]. In this case, daily inpatient assessment revealed no evidence of mania or hypomania, including absence of elevated mood, grandiosity, pressured speech, flight of ideas, increased goal‐directed activity, or psychomotor agitation. There was no association with substance use or medication initiation that would otherwise account for the emergence of symptoms.

Both CSBD and OCD are typically managed in outpatient settings using a combination of psychopharmacologic and psychotherapeutic interventions [1, 19, 37]. This case was therefore unusual in that the patient and his family agreed that inpatient hospitalization was necessary due to significant safety concerns involving both the patient and others. The treatment team faced the challenge of achieving sufficient symptom reduction to allow safe transition to the least restrictive environment, particularly given concerns involving the patient’s mother, who represented his primary and essentially sole residence. This further intensified the urgency of stabilization while also preparing for alternative housing or specialized facilities as the second line option.

Often, CSBD remains underreported and untreated in clinical and research settings, likely due to the shame and stigma of being diagnosed with this condition [38]. The average age of onset is 18.7 years, but most patients do not seek treatment until ~37 years of age, usually due to legal problems, marital conflicts, or professional issues, which limits the availability of data on effective treatments [39, 40]. Despite increasing research on the psychological and neural mechanisms of CSBD and evidence suggesting that therapy is an effective approach to controlling and reducing symptoms of sexual urges and distress in patients, there remains a lack of robust research on the efficacy of pharmacotherapy [39], particularly in acute management and specific populations‐based guidelines.

During inpatient treatment, medication selection was complicated by the constellation of overlapping psychiatric symptoms and underlying neurodevelopmental vulnerabilities. Selective serotonin reuptake inhibitors (SSRIs), which have demonstrated benefit in both OCD and compulsive sexual behavior disorder (CSBD) [7, 22, 37], were therefore titrated and optimized. However, given that the full therapeutic effects of SSRIs typically initially emerge over 2 weeks [22, 41], their immediate utility for acute stabilization was inherently limited.

Fluoxetine has demonstrated effectiveness in reducing hypersexual symptoms, particularly in patients with co‐occurring depression, by increasing central serotonin levels, thereby stabilizing mood, improving sleep, and enhancing energy [32]. Our patient was receiving 20 mg/day of fluoxetine at baseline for mood symptoms and libido reduction. On admission, the dose was titrated to 60 mg/day, the maximum recommended dose. Despite this increase and the addition of carbamazepine, no improvement in sexual impulses was observed.

Risperidone, supported by limited evidence in the context of sexual impulsivity [42], has shown benefit for impulsivity and irritability in autism‐related disorders and IDD and was therefore used as part of a multimodal treatment strategy [43]. Although risperidone may have contributed to mood stabilization and maintenance, clinical observation suggested that naltrexone produced the most meaningful reduction in the frequency and intensity of the patient’s sexual urges. The patient’s transparency regarding his symptoms allowed for close monitoring of treatment response.

Several pharmacologic adjustments occurred in proximity, including up‐titration of fluoxetine and initiation of risperidone, which limits definitive attribution of symptom resolution to naltrexone monotherapy. While the most substantial improvement was associated with the introduction of naltrexone, this case does not permit causal inference regarding the independent effect of any single agent. Nevertheless, the timing of clinical response, the established 2‐week onset of therapeutic effect for fluoxetine, the limited evidence supporting risperidone for compulsive sexual symptoms [22, 41], the patient’s own report of perceived benefit following naltrexone initiation, and prior literature describing rapid responses to naltrexone [30] may support its potential role in acute management. Controlled trials will be necessary to establish more definitive evidence.

A prior study described a patient with CSBD and kleptomania who failed to respond to fluoxetine, behavioral therapy, and psychotherapy but achieved symptom improvement on high‐dose naltrexone (150 mg/day) [28]. An additional report described an individual with major depressive disorder and long‐standing CSBD who experienced remission of compulsive sexual behaviors only after initiation of naltrexone [29].

Naltrexone is primarily indicated for urge‐driven disorders such as alcohol and amphetamine dependence and has also demonstrated efficacy in behavioral addictions, including pathological gambling and kleptomania [29, 44–46]. Neuroimaging and genetic studies suggest that CSBD shares overlapping neurobiological pathways with addiction disorders, particularly within brain regions responsible for reward processing and impulse control [30]. This shared compulsive and reward‐seeking profile may explain why naltrexone, which reduces craving and dampens reinforcement mechanisms, may also benefit individuals with CSBD. One report described a patient with chronic alcohol dependence whose sexual compulsions improved concurrently with reduced alcohol use following initiation of naltrexone [34].

When selecting a treatment regimen, careful consideration of side effect burden and monitoring requirements is essential, particularly in off‐label use. Carbamazepine carries rare but potentially fatal risks of toxic epidermal necrolysis and Stevens‐Johnson syndrome and requires close serum level monitoring due to its narrow therapeutic index, which may compromise adherence [47, 48]. In contrast, although naltrexone carries risks, most serious adverse events are associated with concomitant opioid use or misuse, and isolated complications are comparatively uncommon [49].

This case is noteworthy not only for the potential acute therapeutic role of naltrexone in patients with subthreshold and evolving compulsive sexual and obsessive symptoms but also for the patient’s complex comorbid profile, particularly IDD. IDDs are associated with underrecognized and undertreated psychiatric and medical conditions [50]. Approximately 6%–28% of individuals with intellectual disabilities exhibit inappropriate sexual behaviors, including public masturbation and sexually aggressive behaviors [51]. Despite this prevalence, limited research has examined the intersection of IDD and CSBD or the use of naltrexone in this population [46]. This gap may reflect diagnostic overshadowing, wherein emotional or behavioral symptoms are incorrectly attributed solely to intellectual disability [52]. Accordingly, multidisciplinary assessment and individualized treatment planning are essential for effectively addressing the unique needs of this patient population.

## 4. Conclusion

This case illustrates a severe, diagnostically ambiguous presentation of compulsive sexual symptoms conceptualized as an early CSBD‐like syndrome with overlapping obsessive features in a patient with IDD and ADHD. The clinical course was notable for acute refractoriness to multiple psychotropic optimizations, with the most temporally associated symptomatic improvement observed following initiation of naltrexone. Although the proximity of several concurrent medication adjustments precludes attribution of benefit to naltrexone monotherapy, the rapid improvement highlights the potential relevance of reward‐ and impulse‐modulating interventions in severe subthreshold presentations. More broadly, this case underscores the need for clearer diagnostic frameworks and evidence‐based treatment guidance for the acute management of evolving, comorbid CSBD‐like and sexual obsessive syndromes, particularly in patients with underlying neurodevelopmental conditions.

## Funding

The authors have nothing to report.

## Consent

The patient provided written informed consent for their clinical information to be used in this case report and was informed that any identifying information would be anonymized. The patient understands that this case report may be published and will be used for educational and research purposes. A patient‐signed consent form can be submitted upon request from the reviewer/editor after patient health information is redacted.

## Conflicts of Interest

The authors declare no conflicts of interest.

## Supporting Information

Additional supporting information can be found online in the Supporting Information section.

## Supporting information


**Supporting Information** Institutional case report informed consent form.

## Data Availability

The data supporting this study’s findings are available from the corresponding author upon reasonable request.
