# Supplementary material for: Naltrexone for Acute Management of Compulsive Sexual and Obsessive Symptoms in a Patient With a Neurodevelopmental Disorder
Source: Case Rep Psychiatry. 2026 Jun 9;2026:9442957. doi: 10.1155/crps/9442957 (PMC13247679; doi:10.1155/crps/9442957)
Supplement: Supplementary file 1 — Supporting Information Institutional case report informed consent form. [file CRPS-2026-9442957-s001.docx]

# PATIENT INFORMED CONSENT: CASE REPORT

| Provisional title of the case report: |
| --- |
| I, (write patient’s full name) |
| (address) |
| Have been treated by (Doctor’s name) |
| At Northeast Georgia Medical Center (NGMC) on (dates) |
| If I am not the patient, or the patient is a minor, I confirm that I am legally entitled to give this consent. |

In signing this consent form, I **authorize** this practitioner and other co-authors to write a case report about my treatment.

I understand the following:

- I will not directly benefit from participating in this case report.
- The information that can be shared with other health care professionals, however, may improve the care that is received by others in the future.
- This consent form and the case report will be submitted to the Director of GME Research and Scholarly Activity at Northeast Georgia Medical Center for approval and storage, and for the purpose of future publication of a formal case report.

**Privacy**

Signing this consent form does not remove my rights to privacy**.** My name and protected health information (PHI) will not be shared, unless my authorship is requested and consented, however, I understand that complete anonymity cannot be guaranteed.

Initials

**It is possible that somebody somewhere - for example, somebody who looked after me or a relative - may recognize me.**

My information being used for this case report includes relevant to this case disease history, laboratory findings, photographs and prescribed treatment protocols.

A summary of my treatment will be presented for evaluation by a panel of experts and researchers and may be published in a medical journal under open access license (everybody can see and read such publication freely online) and/or presented at a medical conference, and I give permission for such publication or presentation.

The summary of my treatment and the case report will not contain any personal data. If any personal data are affected, they will be published in anonymized form. Allowing my information to be used in this case report will not involve any additional costs to me. I will not receive any compensation.

My participation is entirely voluntary, and I may withdraw permission to participate in this case report at any time. All my personal data submitted to the GME Research Department will be deleted after notification received from the treating practitioner. However, once the case report is written and published (containing any personal data in anonymized form), it will not be possible for me to withdraw it.

My decision will not result in any penalty or loss of benefits to which I am entitled including the quality of care I receive. The withdrawal can be sent to the treating practitioner (please see contact data above). I may be asked to contribute a “patient’s perspective” on the treatment that I have received.

I can voluntarily decide to provide this perspective or decline.

**By signing this form, I confirm that:**

- The case report has been fully explained to me and all my questions have been answered to my satisfaction
- I have been informed of the risks and benefits, if any, of allowing my information to be used in this case report
- I have been informed that I do not have to participate in this case report
- I have read each page of this form
- I authorize access to my personal health information (medical record) as explained in this form
- I have agreed to participate in this case report

| **Patient or legally entitled caregiver** | |
| --- | --- |
| Patient name | |
| **Signature** | **Name of legal signatory** |
| **Date** | **Signature** |

| **Physician of Record** |
| --- |
| Printed name |
| **Signature** |
| **Date** |

Informed Consent was explained, and this form was administered by the following individual on behalf of the physician of record.

Date

Name

Signature
